# Patient Reported Outcomes Measures Information System (PROMIS) Physical Function and Common Performance‐Based Measures of Function in Patients With Neurologic Conditions in Outpatient Rehabilitation

**DOI:** 10.1002/pri.70159

**Published:** 2026-01-13

**Authors:** Matthew S. Briggs, Brittany Lapin, Yadi Li, Mary Stilphen, Sandra Passek, Christine McDonough, Irene Katzan, Joshua K. Johnson

**Affiliations:** ^1^ Rehabilitation Services, Sports Medicine Research Institute, and Department of Orthopaedics The Ohio State University Wexner Medical Center Columbus Columbus Ohio USA; ^2^ Division of Physical Therapy School of Health and Rehabilitation Sciences The Ohio State University Columbus Ohio USA; ^3^ Department of Quantitative Health Sciences Lerner Research Institute Cleveland Clinic Cleveland Ohio USA; ^4^ Center for Outcomes Research and Evaluation Neurological Institute Cleveland Clinic Cleveland Ohio USA; ^5^ Rehabilitation and Sports Therapy Neurological Institute Cleveland Clinic Cleveland Ohio USA; ^6^ School of Health and Rehabilitation Sciences University of Pittsburgh Pittsburgh Pennsylvania USA; ^7^ Division of Physical Therapy Department of Orthopaedic Surgery Duke University Durham North Carolina USA

**Keywords:** 10‐m Walk Test, 5 time sit‐to‐stand, PROMIS, timed up and go

## Abstract

**Background and Purpose:**

It is unknown how Patient‐Reported Outcomes Measures Information System Physical Function (PROMIS‐PF) corresponds to physical abilities and common performance‐based measures of function in patients with neurologic conditions/disorders in outpatient, ambulatory settings. The purpose of this study was to evaluate the association between PROMIS‐PF and common performance‐based measures of function in patients with neurologic conditions receiving outpatient physical therapy (PT).

**Methods:**

A retrospective analysis of clinical data was conducted from 11 outpatient neurologic PT clinics within a large health care system between 12/2/2019 and 12/30/2022. Adult patients with neurologic conditions who had at least one performance‐based functional measure [Timed up and go (TUG), 5 times sit to stand (5 × STS), and 10‐m walk test (10 MWT)] and one PROMIS‐PF score within 7 days were included. Pearson correlations and linear regression models were used to examine the relationships between the PROMIS‐PF and performance‐based measures.

**Results:**

In our study of 1712 patients (average age 59 (SD 16) years, 44% male, 81% white race), there was a moderate relationship between PROMIS‐PF and TUG and PROMIS‐PF and 5 × STS (*r* = −0.31 and −0.38, respectively; *p* < 0.001). There was a strong association between PROMIS‐PF and 10 MWT (*r* = 0.60; *p* < 0.001). In linear regression models, the variation in PROMIS‐PF explained by the performance measures was the highest for 10 MWT (34.8%), followed by 5 × STS, and TUG (13.5% and 9%, respectively).

**Discussion:**

Results demonstrated moderate associations between PROMIS‐PF and performance‐based measures. Both types of measures provide complementary clinical information for outpatients with neurological conditions.

**Abbreviations**
PROMIS‐PF: patient‐reported outcomes measures information system physical function
PT: physical therapy
TUG: timed up and go
10 MWT: 10‐m walk test
5 × STS: 5 times sit to stand

## 1 Introduction

The routine use of patient‐reported outcome measures is one method of demonstrating and supporting the value, impact, and quality of outpatient rehabilitation (Duncan and Murray 2012; Jette et al. 2009; Weldring and Smith 2013). Patient‐Reported Outcomes Measures Information System Physical Function (PROMIS‐PF) measures self‐reported physical abilities (e.g., physical function) and is a common patient reported outcome measure used in outpatient rehabilitation that is not diagnosis, condition or joint specific (“HealthMeasures: Transforming How Health Is Measured ‐ PROMIS,” 2023; Horn et al. 2021; Ziedas et al. 2022). It has been shown to correlate to other commonly used “legacy” patient reported outcome measures (e.g., Foot and Ankle Ability Measure; Disabilities of the Arm Shoulder and Hand; Knee Injury and Osteoarthritis Score; etc.) (“HealthMeasures: Transforming How Health Is Measured ‐ PROMIS,” 2023; Horn et al. 2021; Ziedas et al. 2022). The PROMIS‐PF has most extensively been examined in patients with orthopedic/musculoskeletal (Alvarez‐Nebreda et al. 2019; Atkinson et al. 2015; Cook et al. 2016; Houck et al. 2020; Karayannis et al. 2023; Kishnani et al. 2024; Liegl et al. 2022; Mizher, Rajan, Kim, Cychosz, Demetracopoulos, and Ellis 2023a) and less in patients with neurologic conditions (Katzan et al. 2016; Lapin et al. 2019).

Further, to provide a more comprehensive understanding of patient outcomes, patient reported outcome measures are often paired with other clinician assessed impairment‐based (e.g., pain, range of motion, muscle strength, etc.) and performance‐based functional measures (e.g., walking tasks, sit‐to‐stand tasks, change of direction task, etc.) (Ageberg et al. 2013; Brodke et al. 2022; Givens et al. 2018; Horn et al. 2020, 2021; Jette et al. 2009; Mizher, Rajan, Kim, Cychosz, Demetracopoulos, and Ellis 2023b; ne Engberg Skaara et al. 2013). Overall, patient reported outcome measures have shown moderate to strong relationships with performance‐based measures of physical function in different populations (Kishnani et al. 2024; Paton et al. 2022). More specifically, the PROMIS‐PF has been shown to have moderate to weak correlations with the performance‐based measures such as the timed‐up‐and‐go, sit to stand, measures of gait speed, and similar tasks in patients with non‐neurologic conditions (Alvarez‐Nebreda et al. 2019; Atkinson et al. 2015; Givens et al. 2018; Houck et al. 2020; Karayannis et al. 2023; Kishnani et al. 2024; Liegl et al. 2022; Mizher, Rajan, Kim, Cychosz, Demetracopoulos, and Ellis 2023a). However it is unknown how PROMIS‐PF corresponds to physical abilities and common performance‐based measures of function in patients with neurologic conditions/disorders in outpatient, ambulatory settings.

Further, to optimize the utility of any tool, it is imperative for those who use it to understand its meaning and how to appropriately interpret the results. Several studies have attempted to improve the understanding and interpretation of the PROMIS‐PF subscale for clinicians, primarily for patients with orthopedic/musculoskeletal conditions (Brodke et al. 2022; Houck et al. 2020). There is a lack of understanding amongst physical therapists who treat patients with neurologic disorders on how to best interpret and utilize the PROMIS‐PF to inform their care. To improve clinician interpretation of PROMIS‐PF, our study aimed to evaluate the relationship between PROMIS‐PF and performance‐based measures of function in a neurologic population receiving outpatient physical therapy (PT). Better understanding of these relationships will provide clinicians with important information on how to best interpret results of the PROMIS‐PF to advance outpatient rehabilitation practice and enhance patient‐centered care management strategies.

## 2 Methods

### 2.1 Study Design and Setting

A retrospective cohort study was conducted including patients who received PT services at any of 11 outpatient neurologic PT clinics within a large health care system between December 2, 2019 and December 30, 2022. The clinics where the data were obtained provide PT services to a large, Midwestern metropolitan area. Records were included for adult patients who had at least one performance‐based functional measure and one PROMIS‐PF score completed within 7 days of one another. The study was approved by Cleveland Clinic Institutional Review Board. Because the study consisted of analyses of pre‐existing data, the requirement for patient informed consent was waived.

### 2.2 Patient Reported Outcomes Measures Information System—Physical Function

Routine clinical care in the outpatient neurologic PT clinics can include completion of PROMIS‐PF computer adaptive test v2.0, developed using the item response theory (Crins et al. 2018). Prior to their clinical visits, the PROMIS‐PF was completed either via MyChart patient portal, or on tablets in the clinic waiting room. The PROMIS‐PF measures self‐reported physical abilities related to the upper (dexterity) and lower extremities (walking or mobility) and spine and measures an individual's ability to perform physical activities from basic self‐care to strenuous task (“HealthMeasures: Transforming How Health Is Measured ‐ PROMIS,” 2023; USER MANUAL AND SCORING INSTRUCTIONS PROMIS PHYSICAL FUNCTION 2024; Horn et al. 2021). The computer adaptive algorithm of the tool dynamically selects questions based on previous responses. This function reduces burden and increases the precision across a variety of symptoms and functional abilities (“HealthMeasures: Transforming How Health Is Measured ‐ PROMIS,” 2023; USER MANUAL AND SCORING INSTRUCTIONS PROMIS PHYSICAL FUNCTION 2024; Horn et al. 2021). Thus, each person completes a different set of questions based on their previous answer and the subsequent questions are adapted based on what the respondent indicates they have problems or difficulty with (e.g., basic self‐care, walking, stairs, balance, household chores, strenuous activity, etc.) and severity of difficulty. The PROMIS‐PF is recommended for most individuals and those with lower extremity concerns versus other PROMIS measures (e.g., PROMIS Mobility or PROMIS Upper Extremity) (USER MANUAL AND SCORING INSTRUCTIONS PROMIS PHYSICAL FUNCTION 2024). The PROMIS‐PF has an item bank of at least 165 items in the adult bank with patients typically answering 4–12 questions based on their responses (USER MANUAL AND SCORING INSTRUCTIONS PROMIS PHYSICAL FUNCTION 2024). The PROMIS‐PF is standardized to a reference general population on a T‐scale with a mean of 50 and standard deviation (SD) of 10 where higher scores indicate better physical functioning. Cut points of ≤ 30, 30–40, 40–45, and ≥ 45 have been suggested for interpreting scores as severe, moderate, mild, or within normal limits (“PROMIS Score Cut Points,” 2024; Rothrock et al. 2019). PROMIS‐PF has been demonstrated as valid and reliable for use in PT and in patients with a variety of chronic, non‐neurological conditions (e.g., osteoarthritis, cardiac disorders), and body regions (e.g., pelvis, hip, leg, knee, lower leg) (Crins et al. 2018).

### 2.3 Performance Based Measures of Lower Extremity Function

The Timed up and go (TUG) (Christopher et al. 2019), 5 times sit to stand (5 × STS) (Goldberg et al. 2012), and 10‐m walk test (10 MWT) (Adell et al. 2013) were selected as performance‐based measures of function as they were the most frequently recorded in the clinics of interest. Therapists chose the performance‐based measures of function that would best correspond and measure the patient's impairments, functional limitations, and diagnosis.

The TUG has been shown to be reliable and valid in assessing individuals ability to ambulate and perform transfers in those with stroke and spinal cord injury (Lam et al. 2008; Pollock et al. 2011). The TUG requires individuals to stand from a chair, walk 3 m, turn 180°, and walk back to the chair and sit back down (Podsiadlo and Richardson 1991; Pollock et al. 2011). The TUG has extensive normative values with faster times (seconds) being indicative of greater function (Bohannon 2006). The TUG has a minimal detectable change score of 2.9 s (Flansbjer et al. 2005).

The 5 × STS is reliable and valid to assess functional mobility with a stand and sit task focusing on lower extremity strength and balance control in a variety of populations including healthy adults, older adults, and those with neurologic conditions (e.g., stroke, vestibular disorders, Parkinson's disease) (Goldberg et al. 2012; Lord et al. 2002; Meretta et al. 2006; Mong et al. 2010; Muñoz‐Bermejo et al. 2021). To perform the assessment, individuals sit in a chair with their arms crossed. They are then instructed to stand up and then return to the sitting position 5 consecutive times as fast as possible. Faster times (seconds) to complete the 5 repetitions is indicative of greater function. The 5 × STS has a minimal clinically important difference of 2.3 s (Meretta et al. 2006).

Finally, the 10 MWT measures the speed at which an individual can walk 10 m (Adell et al. 2013). This test assesses the walking speed in meters per second over a 10‐m distance. The version of the test that assessed the self‐selected walking speed was selected for this investigation. The standard protocol used by the clinicians involved measuring the time with a stopwatch (in seconds) for the patients to walk the middle 6‐m of the 10‐m distance. The 6‐m was then divided by the time taken to complete that distance. An assistive device (e.g., cane) may be used for this test. This test has been shown to be reliable and valid in a variety of populations including healthy adults, older adults, and those with neurologic conditions (e.g., spinal cord injury, traumatic brain injury, Parkinson's disease) (Bowden and Behrman 2007; Hirsch et al. 2014; Kim et al. 2016; Lang et al. 2016; Peters et al. 2013; Scivoletto et al. 2011; Van Hedel et al. 2006) The 10 MWT has a minimal clinically important difference of 0.05 m/s (Perera et al. 2006).

### 2.4 Data Collection

Patient and clinical data were extracted from the electronic medical records by the authors (JJ and YL). Data obtained included attendance status (e.g., data of initial evaluation, date of episode discontinuation of care, number of patient visits), sociodemographic variables (e.g., age, gender, race, ethnicity, employment status, highest level of education, median household income, insurance provider), PROMIS‐PF scores, and performance measures. The “PT treatment diagnoses” were also included in the data extraction (Tables S1 and S2).

### 2.5 Statistical Analysis

Demographics, PROMIS‐PF, and performance measures were summarized using descriptive statistics. Selection bias was assessed by comparing characteristics of patients included (for having performance measures and PROMIS‐PF completed within 7 days) with those excluded from the study. Categorical variables were compared using the chi‐square test, while continuous variables were compared using *t*‐test or Wilcoxon Rank Sum test.

The association between PROMIS‐PF and performance measures was evaluated using Pearson correlation coefficient with 95% confidence intervals where 0.1–0.3, 0.3–0.5, and > 0.5 indicate weak, moderate, and strong associations (Cohen 1988). Additionally, linear regression models were constructed to predict PROMIS‐PF including the performance measures in three separate models and adjusted for demographics chosen a priori based on clinical relevance: age, sex, race, marital status, and median household income. The variation of PROMIS‐PF explained by each performance measure was evaluated by calculating the change in *R* ^2^ in the model without and with the performance measure. Linear regression assumptions including normality of residuals and no multicollinearity were met by checking the diagnostic plot, correlation matrix and variance inflation factor.

In addition, we developed a protype figure to illustrate and better communicate these relationships to clinicians. Average performance measures were compared across strata of PROMIS‐PF T‐score categories: within normal limits (> 45), mild (40–45), moderate (30–40), and severe (≤ 30) symptoms. All analyses were conducted using SAS 9.4 statistical software (SAS Inc., Cary, NC). Statistical significance was established throughout at *p* < 0.05.

## 3 Results/Findings

**FIGURE 1:**
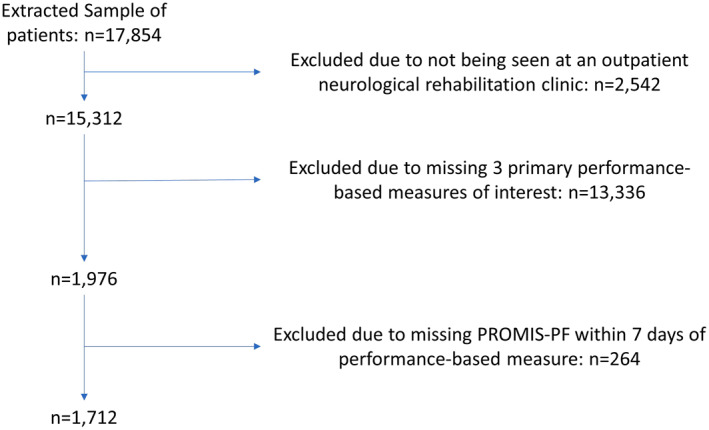
Flowchart illustrating the original extracted clinical data. Several clinics that were included in the original data extraction did not focus on neurologic rehabilitation and were subsequently excluded (*n* = 2542). Next, patients were excluded (*n* = 13,336) if they were missing the 3 primary performance‐based measures (timed up and go, 5 times sit‐to‐stand, and 10‐m walk test). Finally, patients were excluded (*n* = 264) from the final data set if their PROMIS‐PF was not within 7 days of the collected performance‐based measure. A total of 1712 patients were included in the data set for the final analysis.

There were 17,854 patients who received physical therapy services at outpatient neurologic PT clinics between December 2, 2019 and December 30, 2022 (Figure 1). Of these, 1712 had one of the three most common performance measures, PROMIS‐PF and were included in our study. Included patients (average age 59.2 ± 15.6) were of similar age to those excluded, but more likely to be male (43.8% vs. 34.8%), of white race (81.5% vs. 75.1%), married (61.2% vs. 51.4%), and have higher household income compared to excluded patients (Table 1). Patients included in the study also had significantly worse PROMIS‐PF scores as compared to patients excluded (37.9 ± 7.9 vs. 41.1 ± 9.2), with significantly more patients in the moderate and severe categories (Table 1). Although not statistically significant, patients included in the study had clinically meaningfully better scores on the TUG and on the 10 MWT as compared to those who were excluded. However, patients included in the study had statistically significant slower times during the 5 × STS test than those excluded (difference: 2 s), but this was not clinically meaningful (Table 1).

**TABLE 1:** Patient characteristics (*N* = 17,854), stratified by included versus not included in the analyses.

	Overall (*N* = 17,854)		Included in analysis (*N* = 1712)		Excluded (*N* = 16,142)		*p*‐value
	*N*	Statistics	*N*	Statistics	*N*	Statistics	
Age	17,854	59.6 (17.5)	1712	59.2 (15.6)	16,142	59.6 (17.7)	0.30^a2^
Gender	17,854		1712		16,142		***<*** ***0*.*001*** ^***d***^
Female		11,490 (64.4)		962 (56.2)		10,528 (65.2)	
Male		6360 (35.6)		750 (43.8)		5610 (34.8)	
Other		4 (0.02)		0 (0.00)		4 (0.02)	
Race	17,408		1677		15,731		***<*** ***0*.*001*** ^***c***^
White		13,182 (75.7)		1367 (81.5)		11,815 (75.1)	
Black		3358 (19.3)		239 (14.3)		3119 (19.8)	
Other		868 (5.0)		71 (4.2)		797 (5.1)	
Ethnic group	17,401		1656		15,745		***0*.*024*** ^***c***^
Hispanic		658 (3.8)		46 (2.8)		612 (3.9)	
Not Hispanic		16,743 (96.2)		1610 (97.2)		15,133 (96.1)	
Marital status	17,667		1703		15,964		***<*** ***0*.*001*** ^***c***^
Married		9251 (52.4)		1043 (61.2)		8208 (51.4)	
Single		5185 (29.3)		409 (24.0)		4776 (29.9)	
Other		3231 (18.3)		251 (14.7)		2980 (18.7)	
Employment status	17,369		1634		15,735		***0*.*026*** ^***c***^
Full time		4559 (26.2)		397 (24.3)		4162 (26.5)	
Not employed		6793 (39.1)		630 (38.6)		6163 (39.2)	
Retired		4701 (27.1)		491 (30.0)		4210 (26.8)	
Other		1316 (7.6)		116 (7.1)		1200 (7.6)	
Median household income	17,723	53,107 [41,651, 66,659]	1703	54,722 [43,134, 66,047]	16,020	53,107 [41,651, 67,007]	***0*.*002*** ^***b***^
PROMIS physical function T‐score	9734	40.5 (9.0)	1712	37.9 (7.9)	8022	41.1 (9.2)	***<*** ***0*.*001*** ^***a2***^
PROMIS physical function category	9734		1712		8022		***<*** ***0*.*001*** ^***c***^
Normal (> 45)		2792 (28.7)		289 (16.9)		2503 (31.2)	
Mild (40–45)		1996 (20.5)		301 (17.6)		1695 (21.1)	
Moderate (30–40)		3671 (37.7)		818 (47.8)		2853 (35.6)	
Severe (≤ 30)		1275 (13.1)		304 (17.8)		971 (12.1)	
Performance measures							
Timed up and go (s)	1818	18.4 (25.5)	1563	17.8 (22.9)	255	22.2 (37.5)	0.068^a2^
5 times sit to stand (s)	1311	18.1 (12.0)	1121	18.4 (12.5)	190	16.4 (8.8)	***0*.*006*** ^***a2***^
10‐m walk test (m/s)	1137	0.88 (0.34)	956	0.89 (0.34)	181	0.84 (0.36)	0.11^a1^

*Note:* Statistics presented as Mean (SD), Median [P25, P75], *N* (column %). *p*‐values: a1 = *t*‐test, a2 = Satterthwaite *t*‐test, b = Wilcoxon Rank Sum test, c = Pearson's chi‐square test, d = Fisher's Exact test. Bold values indicate statistical significance was reached (*p* < 0.05).

The association between PROMIS‐PF and TUG and 5 × STS was moderate (*r* = −0.31 and −0.38, respectively), while the association between PROMIS‐PF and 10 m gait speed was strong (*r* = 0.60) (Table 2). All three performance measures were significantly associated with PROMIS‐PF (Table 3). The variation in PROMIS‐PF explained by the performance measures was the highest for 10 MWT gait speed (34.8%), followed by 5 × STS and TUG (13.5% and 9%, respectively). The full model results are presented in Table S3.

**TABLE 2:** Pearson correlation coefficient between performance measures and PROMIS physical function.

	*N*	Correlation coefficients (95% CI)	*p*‐value
Performance measures			
Timed up and go (s)	1563	−0.31 (−0.35, −0.26)	***<*** ***0*.*001***
5 times sit to stand (s)	1121	−0.38 (−0.43, −0.33)	***<*** ***0*.*001***
10‐m walk test (m/s)	956	0.60 (0.56, 0.64)	***<*** ***0*.*001***

*Note:* Bold values indicate statistical significance was reached (*p* < 0.05).

**TABLE 3:** Linear regression models predicting PROMIS physical function.

	*N* Used	Estimate (95% CI)	*p*‐value	Adjusted *R* ^2^ in model without performance measure	Adjusted *R* ^2^ in model with only performance measure	Adjusted *R* ^2^ in model with demographics and performance measure
Timed up and go (s)	1519	−0.10 (0.01)	***<*** ***0*.*001***	0.011	0.090	0.101
5 times sit to stand (s)	1090	−0.24 (0.02)	***<*** ***0*.*001***	0.009	0.135	0.144
10‐m walk test (m/s)	934	14.22 (0.63)	***<*** ***0*.*001***	0.013	0.348	0.361

*Note:* Each row represents a separate multivariable linear regression model adjusted for age, sex, race, marital status, and median household income. Bold values indicate statistical significance was reached (*p* < 0.05).

**FIGURE 2:**
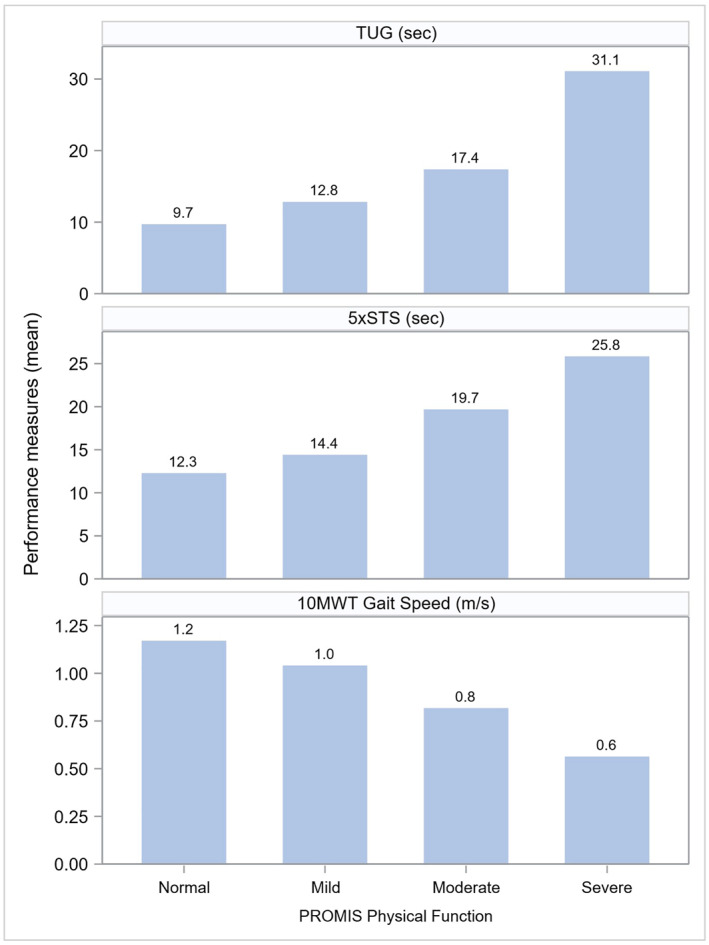
Bar chart of performance measures, by PROMIS physical function categories. Footnote: PROMIS physical function *T*‐score categories: Normal (> 45), Mild (40–45), Moderate (30–40), Severe (≤ 30) symptoms.

Figure 2 presents a visual interpretation of the average performance measures stratified by PROMIS‐PF category. It illustrates the relationships between the performance measures and self‐reported normal, mild, moderate, and severe physical function scores.

## 4 Discussion

Our results indicate overall moderate associations between PROMIS‐PF scores and the TUG, 5 × STS, and 10 MWT in patients with neurologic conditions receiving outpatient PT care. These findings advance previous findings showing similar moderate to weak correlations with the performance‐based measures such as the STS tests, TUG, measures of gait speed, and similar tasks in a variety of patients with non‐neurologic conditions (e.g., ankle arthroplasty, chronic low back pain, fractures, cardiovascular conditions, cancer, etc.) (Alvarez‐Nebreda et al. 2019; Atkinson et al. 2015; Houck et al. 2020; Karayannis et al. 2023; Liegl et al. 2022; Mizher, Rajan, Kim, Cychosz, Demetracopoulos, and Ellis 2023a). When examining the three performance‐based measures of function in our sample, gait speed (e.g., 10 MWT), showed the strongest association with PROMIS‐PF scores. This is contrary to findings in individuals with low back pain showing weak to no associations with various measures of walking (e.g., 2‐min walk, 10 MWT) (Karayannis et al. 2023). The divergence in findings is potentially due to functional limitations that may be different in those with neurologic conditions versus those with low back pain and the questions answered on the PROMIS‐PF would likely be different.

Although we were unable to perform diagnosis‐specific analysis for different diagnostic subgroups (Tables S1 and S2) in this study, future work should examine these potential relationships. For example, the diagnosis group of “unspecified gait/mobility conditions” was the most prevalent diagnosis group in our patient sample (Tables S1 and S2). As such, patients in our study were likely more aware of limitations related to walking. This perhaps had a stronger influence on how patients responded to walking and non‐walking related questions on the PROMIS‐PF. Further, the strength of associations between the PROMIS‐PF may be different in patients with other neurologic diagnoses with limitations primarily related to “balance/coordination” impairments or “dizziness” (Tables S1 and S2). For example, the TUG requires considerable balance and coordination due to the different components required of the task (e.g., standing, walking, turning, and sitting down) and patients may have responded differently on the PROMIS‐PF than patients with other diagnoses (Nightingale et al. 2019; Podsiadlo and Richardson 1991; Pollock et al. 2011). Further, patients with neurologic diagnoses related to “weakness” or “fatigue” may answer questions differently on the PROMIS‐PF, which in turn may influence the relationship of the PROMIS‐PF score with the 5 × STS task which focuses more on lower extremity strength (Goldberg et al. 2012; Lord et al. 2002; Mentiplay et al. 2020; Meretta et al. 2006; Mong et al. 2010; Muñoz‐Bermejo et al. 2021). However, each of the performance‐based functional measures used in this study have been used to assess a variety of different functional constructs together (e.g., mobility, strength, balance, etc.). As such, the aforementioned discussion is just speculation and should be explicitly examined in future work.

Ultimately, our findings in patients with neurologic conditions support and build upon previous literature (Alvarez‐Nebreda et al. 2019; Atkinson et al. 2015; Houck et al. 2020; Karayannis et al. 2023; Liegl et al. 2022; Mizher, Rajan, Kim, Cychosz, Demetracopoulos, and Ellis 2023a), suggesting that the PROMIS‐PF and the performance‐based measures of function do not provide clinicians with redundant information. Similar to the conclusions of (Christensen et al. 2022), both patient‐reported outcomes and performance‐based measures measuring lower extremity function (e.g., mobility, gait speed, strength, balance, etc.) should be used to fully characterize the physical function of patients and be used concurrently to more comprehensively understand patients' function. Thus, each clinical element, whether subjectively reported by the patient or objectively measured by performance, gives clinicians insight into two different—but equally important—constructs. Clinicians might consider any difference between the PROMIS‐PF score and observed scores on performance‐based measures (e.g., TUG, 5 × STS, 10 MWT, etc.) as an opportunity for discussion and shared decision‐making about the rehabilitation care plan.

Furthermore, improving clinician and patient interpretation of patient reported outcomes, such as the PROMIS‐PF, is imperative in the promotion of patient‐centered care (Houck et al. 2020; Keeney et al. 2022; Kotronoulas et al. 2014). We provide a figure (Figure 2) which may assist clinicians aligning PROMIS‐PF scores with the TUG, 5 STS, and 10 MWT in patients with neurologic conditions. Moreover, self‐reported physical function may also be affected by individual patient characteristics, including self‐efficacy and mental health (Gruber‐Baldini et al. 2017; Jacobson et al. 2020). Although our study did not examine these factors, including such measures may help to further characterize patients and promote individualized patient care. There is a need to continue to examine how the PROMIS‐PF may help to inform clinicians in their care and management of patients with neurologic conditions in outpatient physical therapy.

## 5 Limitations and Future Research

Although our study has many strengths, including a large sample of patients who completed PROMIS‐PF as standard care, there are some limitations that warrant discussion. Most patients in the initial data pull did not have PROMIS‐PF and performance‐based measures collected within 7 days of one another. Currently, in the health system used in the study, patients complete PROMIS‐PF at 28‐day intervals. It may be appropriate to collect these measures at more frequent intervals, and future research should evaluate timing in the outpatient setting. Furthermore, due to the pragmatic and clinical nature of the data collection, it is unknown whether patients completed the PROMIS‐PF in a supervised or unsupervised setting, as it could be completed in various locations. Additionally, we could not evaluate the impact of limited digital literacy, cognitive ability, or vision on the completion of the PROMIS‐PF. It is likely that individuals with these limitations did not complete PROMIS‐PF and therefore not represented in the sample. Future studies should attempt to control for these factors. Patients included in our study were also more likely to be male, white, married, retired, and having higher household income, which could limit the generalizability of our findings. The “PT treatment diagnosis” included in this study was diverse and based on referring ICD‐10 codes (Tables S1 and S2) and reflective of what the physical therapist would focus on in their treatment and plan of care. Additional investigation is necessary to better understand how PROMIS‐PF scores may vary based on more diverse patient demographics (e.g., race, ethnicity, socio‐economic status, geographic location, etc.) than those included in this project. Further, due to the nature of the data available to us, it was not feasible to collect specific physical examination data such as motor palsy or sensory impairment. Examining the relationships amongst PROMIS‐PF scores and performance‐based measures of function in patients with distinct neurologic diagnoses and impairments may enhance specificity and individualization of treatment. Furthermore, the performance‐based measure of function used in this study (e.g., TUG, 5 × STS, 10 MWT) primarily focused on constructs of lower extremity function and mobility as these were chosen by the treating clinicians based on the patients' impairments, functional limitations, and diagnosis. Examining the relationships of the PROMIS‐PF with performance‐based measures focused on the upper extremity or the spine in patients with neurologic conditions should also be examined. Finally, it is crucial for future studies to examine the utilization, interpretation, and perceived value of the PROMIS‐PF in both clinicians and patients with neurologic conditions to further promote patient‐centered care.

## 6 Implications on Physiotherapy Practice

While the PROMIS‐PF subscale provides important information related to patients' perception of their physical function, moderate associations with performance‐based measures of lower extremity function in patients with neurologic conditions were seen in an outpatient setting. Thus, utilizing both types of measures is important in creating a comprehensive picture and profile of patients with neurologic conditions. Results from this study offer opportunities to further examine and understand the value of the PROMIS‐PF subscale in patients with neurological conditions while promoting patient‐centered care.

## Author Contributions

Matthew Briggs is the lead author and oversaw all aspects of the study and was intimately involved in the coordination, preparation, data collection, analysis, interpretation, and development, revisions, and final approval of the manuscript. Brittany Lapin and Yadi Li provided substantial statistical support and results interpretation and were involved in all aspects of the development, revisions, and final approval of the manuscript. Mary Stilphen, Sandra Passek, Christine McDonough, Irene Katzan, and Joshua K. Johnson provided unique operational and content expertise on the topics presented in the manuscript. They provided input during the initial planning stages, study execution, and result interpretation. They were directly involved in the development, revisions, and final approval of the manuscript.

## Funding

Briggs: Received funding from the Learning Health Systems Rehabilitation Research Network (which is supported by the National Institutes of Health under award number 1P2CHD101895‐01 through the Eunice Kennedy Shriver National Institute of Child Health and Human Development and the National Institute of Nursing Research) as a Learning Health Systems Scholar for the conduct of this study; Lapin: None; Li: None; Stilphen: None; Passek: None; McDonough: None; Katzan: None; Johnson: None.

## Ethics Statement

The study was approved by the Cleveland Clinic Institutional Review Board and determined to be Exempt Human Subject Research (IRB# 22–1128).

## Consent

The authors have nothing to report.

## Conflicts of Interest

The authors declare no conflicts of interest.

## Supporting information


**Table S1**: Frequency and percentage of PT treatment diagnosis.

**Table S2**: Details of specific PT treatment diagnosis name.

**Table S3**: Linear regression models predicting PROMIS physical function (full model results).

## Data Availability

The data that support the findings of this study are available on request from the corresponding author. The data are not publicly available due to privacy or ethical restrictions.
