# Supplementary material for: Patient Reported Outcomes Measures Information System (PROMIS) Physical Function and Common Performance‐Based Measures of Function in Patients With Neurologic Conditions in Outpatient Rehabilitation
Source: Physiother Res Int. 2026 Jan 13;31(1):e70159. doi: 10.1002/pri.70159 (PMC12797007; doi:10.1002/pri.70159)
Supplement: Supplementary file 1 — Table S1: Frequency and percentage of PT treatment diagnosis. [file PRI-31-e70159-s001.docx]

**Table S1. Frequency and percentage of PT treatment diagnosis**

| **Diagnosis** | **N (%)** |
| --- | --- |
| Unspecified gait/mobility conditions | 669 (39.08) |
| Known progressive sensorimotor disease | 424 (24.77) |
| Unspecified balance/coordination conditions | 235 (13.73) |
| Functional neurological symptom disorders | 126 (7.36) |
| Dizziness, with or without vestibular cause | 65 (3.8) |
| Weakness/tremor | 55 (3.21) |
| Ischemic or hemorrhagic stroke | 39 (2.28) |
| Spasticity | 33 (1.93) |
| Post-acute sequelae of COVID-19 (PASC) | 22 (1.29) |
| Fatigue | 9 (0.53) |
| Cognitive impairment | 8 (0.47) |
| Headaches/migraine | 8 (0.47) |
| Neuro-Oncologic conditions | 7 (0.41) |
| Traumatic brain or spinal cord injury | 6 (0.35) |
| Depression/anxiety | 3 (0.18) |
| Non-stroke vascular conditions | 3 (0.18) |
