# Supplementary material for: Patient Reported Outcomes Measures Information System (PROMIS) Physical Function and Common Performance‐Based Measures of Function in Patients With Neurologic Conditions in Outpatient Rehabilitation
Source: Physiother Res Int. 2026 Jan 13;31(1):e70159. doi: 10.1002/pri.70159 (PMC12797007; doi:10.1002/pri.70159)
Supplement: Supplementary file 2 — Table S2: Details of specific PT treatment diagnosis name. [file PRI-31-e70159-s002.docx]

**Table S2. Details of specific PT treatment diagnosis name**

| **Diagnosis** | **Specific diagnosis name** |
| --- | --- |
| Unspecified gait/mobility conditions | Abnormality of gait |
|  | Abnormality of gait and mobility |
|  | Abnormality of gait due to impairment of balance |
|  | Gait abnormality |
|  | Unsteady gait |
|  | Neurodegenerative gait disorder |
|  | Neurologic gait disorder |
|  | Neurologic gait dysfunction |
|  | Multifactorial gait disorder |
|  | Gait difficulty |
|  | Gait disorder |
|  | Gait disturbance |
|  | Gait instability |
|  | Impaired gait |
|  | Impaired gait and mobility |
|  | Impaired functional mobility, balance, and endurance |
|  | Impaired functional mobility, balance, gait, and endurance |
|  | Decreased functional mobility |
|  | Decreased functional mobility and endurance |
|  | Decreased strength, endurance, and mobility |
|  | Impaired mobility |
|  | Difficulty walking |
|  | Decreased activities of daily living (ADL) |
|  | Impaired instrumental activities of daily living (IADL) |
|  | Alteration in self-care ability |
|  | Abnormal involuntary movements |
|  | Apraxia |
|  | Gross motor impairment |
|  | Impaired transfers |
| Known progressive sensorimotor disease | Multiple sclerosis (HCC) |
|  | Multiple sclerosis exacerbation (HCC) |
|  | Multiple sclerosis, relapsing-remitting (HCC) |
|  | Myasthenia gravis (HCC) |
|  | Myasthenia gravis without exacerbation (HCC) |
|  | Parkinson's disease (HCC) |
|  | Parkinsonian features |
|  | Parkinsonism, unspecified Parkinsonism type (HCC) |
|  | Primary parkinsonism (HCC) |
|  | Vascular parkinsonism (HCC) |
|  | Cerebellar ataxia (HCC) |
|  | Cerebral palsy, unspecified type (HCC) |
|  | Infantile cerebral palsy (HCC) |
|  | ALS (amyotrophic lateral sclerosis) (HCC) |
|  | Axonal neuropathy |
|  | Demyelinating disease of central nervous system (HCC) |
|  | Fibromyalgia |
|  | Motor neuron disease (HCC) |
|  | Seizure (HCC) |
|  | Transverse myelitis (HCC) |
| Unspecified balance/coordination conditions | Balance disorder |
|  | Balance problem |
|  | Imbalance |
|  | Loss of balance |
|  | Impairment of balance |
|  | Unstable balance |
|  | Decreased coordination |
|  | Lack of coordination |
|  | Ataxia |
|  | Ataxic gait |
|  | Unsteadiness on feet |
|  | Risk for falls |
|  | At high risk for injury related to fall |
|  | At risk for falls |
|  | Fall, initial encounter |
|  | Fall, subsequent encounter |
|  | Recurrent falls |
|  | Frequent falls |
|  | Cervicocranial syndrome |
|  | Dyskinesia |
| Functional neurological symptom disorders | Functional neurologic complaint |
|  | Functional neurological symptom disorder with abnormal movement |
|  | Functional neurological symptom disorder with mixed symptoms |
|  | Functional neurological symptom disorder with weakness or paralysis |
|  | Focal dystonia |
|  | Functional movement disorder |
| Dizziness, with or without vestibular cause | Dizziness |
|  | Dizziness and giddiness |
|  | Vertigo |
|  | Vertigo of central origin |
|  | BPPV (benign paroxysmal positional vertigo), left |
|  | BPPV (benign paroxysmal positional vertigo), right |
|  | Benign paroxysmal positional vertigo, unspecified laterality |
|  | Disorder of vestibular function of both ears |
|  | Syncope and collapse |
| Weakness/tremor | Weakness |
|  | Generalized weakness |
|  | Bilateral leg weakness |
|  | Right sided weakness |
|  | Foot drop, bilateral |
|  | Bilateral foot-drop |
|  | Foot drop, right |
|  | Tremor |
|  | Tremors of nervous system |
|  | Action tremor |
|  | Functional tremor |
| Ischemic or hemorrhagic stroke | Cerebrovascular accident (CVA) due to embolism of right anterior cerebral artery (HCC) |
|  | Cerebrovascular accident (CVA) due to occlusion of left middle cerebral artery (HCC) |
|  | Cerebrovascular accident (CVA), unspecified mechanism (HCC) |
|  | Acute CVA (cerebrovascular accident) (HCC) |
|  | History of CVA (cerebrovascular accident) |
|  | H/O: CVA (cerebrovascular accident) |
|  | Late effects of CVA (cerebrovascular accident) |
|  | Arterial ischemic stroke (HCC) |
|  | Arterial ischemic stroke, MCA (middle cerebral artery), left, acute (HCC) |
|  | Arterial ischemic stroke, PCA (posterior cerebral artery), left, acute (HCC) |
|  | Acute ischemic stroke (HCC) |
|  | SAH (subarachnoid hemorrhage) (HCC) |
|  | Subarachnoid hemorrhage (HCC) |
|  | Subarachnoid bleed (HCC) |
|  | Intracranial hemorrhage (HCC) |
|  | Lacunar stroke (HCC) |
|  | Right thalamic stroke (HCC) |
|  | History of stroke |
| Spasticity | Spasticity |
|  | Spastic diplegic cerebral palsy (HCC) |
|  | Spastic hemiparesis (HCC) |
|  | Spastic hemiplegia of left nondominant side due to noncerebrovascular etiology (HCC) |
|  | Spastic hemiplegia of right dominant side as late effect of cerebral infarction (HCC) |
| Post-acute sequelae of COVID-19 (PASC) | History of COVID-19 |
|  | Post-acute sequelae of COVID-19 (PASC) |
| Fatigue | Chronic fatigue syndrome |
|  | Fatigue, unspecified type |
| Cognitive impairment | MCI (mild cognitive impairment) |
|  | Mild cognitive impairment |
|  | Impaired cognition |
|  | Alzheimer's disease (HCC) |
|  | Lewy body dementia without behavioral disturbance (HCC) |
| Headaches/migraine | Headaches |
|  | Migraine with aura, not intractable, without status migrainosus |
|  | Chronic migraine without aura, with intractable migraine, so stated, with status migrainosus |
|  | Intractable migraine with aura without status migrainosus |
|  | Intractable chronic migraine without aura and without status migrainosus |
| Neuro-Oncologic conditions | Astrocytoma brain tumor (HCC) |
|  | Brain tumor (HCC) |
|  | Brain lesion |
|  | Meningioma (HCC) |
|  | Vestibular schwannoma (HCC) |
|  | White matter abnormality on MRI of brain |
| Traumatic brain or spinal cord injury | Motor vehicle accident, subsequent encounter |
|  | Paraplegia (HCC) |
|  | Post concussion syndrome |
|  | Quadriparesis (HCC) |
| Depression/anxiety | Depressed mood |
|  | Generalized anxiety disorder |
|  | Recurrent major depression in partial remission (HCC) |
| Non-stroke vascular conditions | TIA (transient ischemic attack) |
|  | Unruptured cerebral aneurysm |
|  | Vertebrobasilar artery syndrome |
