# Supplementary material for: Patient Reported Outcomes Measures Information System (PROMIS) Physical Function and Common Performance‐Based Measures of Function in Patients With Neurologic Conditions in Outpatient Rehabilitation
Source: Physiother Res Int. 2026 Jan 13;31(1):e70159. doi: 10.1002/pri.70159 (PMC12797007; doi:10.1002/pri.70159)
Supplement: Supplementary file 3 — Table S3: Linear regression models predicting PROMIS physical function (full model results). [file PRI-31-e70159-s003.docx]

**Table S3. Linear regression models predicting PROMIS Physical Function (full model results)**

|  | **Model without performance measure** | | **Model with performance measure** | |
| --- | --- | --- | --- | --- |
|  | **Estimate (95% CI)** | **p-value** | **Estimate (95% CI)** | **p-value** |
| **Performance measure: Timed up and go** |  |  |  |  |
| Age | -0.03 (0.01) | ***0.017*** | -0.02 (0.01) | 0.23 |
| Female | -0.87 (0.42) | ***0.038*** | -0.71 (0.40) | 0.074 |
| Race (reference=White) |  |  |  |  |
| Black | 0.43 (0.63) | 0.50 | 1.06 (0.60) | 0.080 |
| Other | -0.14 (1.02) | 0.89 | -0.18 (0.97) | 0.85 |
| Marital Status (reference=Married) |  |  |  |  |
| Single | -1.70 (0.53) | ***0.002*** | -1.42 (0.51) | ***0.006*** |
| Other | -1.44 (0.59) | ***0.015*** | -1.29 (0.57) | ***0.022*** |
| Median household income (per $10k) | 0.14 (0.11) | 0.21 | 0.08 (0.11) | 0.44 |
| Timed up and go (s) | - | - | -0.10 (0.01) | ***<0.001*** |
| **Performance measure: 5 time sit to stand** |  |  |  |  |
| Age | -0.05 (0.02) | ***0.010*** | -0.03 (0.02) | 0.11 |
| Female | -0.90 (0.51) | 0.078 | -0.88 (0.47) | 0.063 |
| Race (reference=White) |  |  |  |  |
| Black | 0.29 (0.76) | 0.70 | 1.12 (0.71) | 0.12 |
| Other | -0.89 (1.28) | 0.49 | -0.53 (1.19) | 0.65 |
| Marital Status (reference=Married) |  |  |  |  |
| Single | -1.22 (0.67) | 0.067 | -0.93 (0.62) | 0.13 |
| Other | -1.53 (0.72) | ***0.035*** | -0.85 (0.67) | 0.21 |
| Median household income (per $10k) | 0.18 (0.14) | 0.18 | 0.01 (0.13) | 0.95 |
| 5 time sit to stand (s) | - | - | -0.24 (0.02) | ***<0.001*** |
| **Performance measure: 10-meter walk test** |  |  |  |  |
| Age | -0.05 (0.02) | ***0.007*** | 0.01 (0.02) | 0.36 |
| Female | -0.84 (0.53) | 0.11 | 0.08 (0.43) | 0.85 |
| Race (reference=White) |  |  |  |  |
| Black | 0.42 (0.81) | 0.60 | 2.36 (0.66) | ***<0.001*** |
| Other | 1.38 (1.40) | 0.32 | 1.00 (1.12) | 0.37 |
| Marital Status (reference=Married) |  |  |  |  |
| Single | -1.18 (0.70) | 0.092 | 0.07 (0.56) | 0.90 |
| Other | -0.98 (0.76) | 0.20 | -0.28 (0.61) | 0.64 |
| Median household income (per $10k) | 0.36 (0.14) | ***0.010*** | 0.06 (0.11) | 0.59 |
| 10-meter walk test (m/s) | - | - | 14.22 (0.63) | ***<0.001*** |
